# Genome-Wide Analysis of HECT E3 Ligases Members in *Phyllostachys edulis* Provides Insights into the Role of *PeHECT1* in Plant Abiotic Stress Response

**DOI:** 10.3390/ijms252211896

**Published:** 2024-11-05

**Authors:** Xinru Xie, Songping Hu, Linxiu Liu, Huanhuan Pan, Hu Huang, Xun Cao, Guirong Qiao, Xiaojiao Han, Wenmin Qiu, Zhuchou Lu, Renying Zhuo, Jing Xu

**Affiliations:** 1 State Key Laboratory of Tree Genetics and Breeding, Research Institute of Subtropical Forestry, Chinese Academy of Forestry, Hangzhou 311400, China; xie2676527718@163.com (X.X.); lxliu9968@163.com (L.L.); phuanhuan2023@163.com (H.P.); 15272978041@163.com (H.H.); 16671050824@163.com (X.C.); gr_q1982@163.com (G.Q.); hanxj@caf.ac.cn (X.H.); qiuwm05@163.com (W.Q.); luzc@caf.ac.cn (Z.L.); zhuory@gmail.com (R.Z.); 2 College of Bioscience and Bioengineering, Jiangxi Agricultural University, Nanchang 330045, China; sp6974@163.com

**Keywords:** moso bamboo (*Phyllostachys edulis*), HECT E3 ubiquitin ligases, gene family, abiotic stresses

## Abstract

Homology to E6-AP Carboxy Terminus (HECT) E3 ubiquitin ligases play pivotal roles in plant growth, development, and responses to abiotic stresses. However, the function of HECT genes in *Phyllostachys edulis* (*P. edulis*) remains largely uninvestigated. In this study, a comprehensive genome-wide analysis of the HECT E3 ubiquitin ligases gene family in *P. edulis* was conducted, aiming to elucidate its evolutionary relationships and gene expansion. Analysis of gene structure, conserved motifs and domains, and synteny genome regions were performed. Furthermore, cis-elements in HECT gene promoters that respond to plant hormones and environmental stresses were identified and corroborated by expression data from diverse abiotic stress conditions and hormone treatments. Based on the co-expression network of PeHECTs under cold and dehydration stresses, *PeHECT1* was identified as a key candidate gene associated with abiotic stress tolerance. Overexpression of *PeHECT1* in tobacco leaves significantly upregulated genes related to reactive oxygen species (ROS) detoxification and polyamine biosynthesis. Yeast one-hybrid (Y1H), electrophoretic mobility shift assay (EMSA), and dual-luciferase (dual-LUC) assays suggested that the transcription factor ETHYLENE RESPONSE FACTOR 3 (PeERF3) bound to the dehydration-responsive element (DRE) of the promoter of *PeHECT1* and activated its transcription activity. Phylogenetic analysis indicated that PeHECT1 in *P. edulis* exhibited a close association with the diploid herbaceous bamboo *Olyra latifolia*, followed by the divergence of rice and bamboo. In summary, this study enhances our comprehensive understanding of the HECT E3 ubiquitin ligases gene family in *P. edulis* and highlights the potential role of *PeHECT1* in plant abiotic stress response.

## 1. Introduction

*Phyllostachys edulis* (*P. edulis*), commonly known as moso bamboo, is a prevalent species in both tropical and subtropical forests, renowned for its rapid growth and remarkable specific strength. Due to its multifaceted uses in food production, fiber, and various other commodities, bamboo plays a critical economic role, especially in East Asia countries. Throughout its life cycle, bamboos regularly encounter numerous abiotic stresses, including extreme temperature fluctuations, drought, and salinity, which significantly hinder bamboo’s growth, development, quality, and yield. In response, plants have developed a range of adaptive mechanisms at the molecular, physiological, biochemical, and cellular levels to mitigate the adverse effects of these abiotic stressors [1,2].

Ubiquitination, a significant post-translational modification in eukaryotes, is essential for the regulation of protein stability and activity, significantly influencing plant growth, development, and responses to abiotic stress [3,4,5]. A distinctive feature of ubiquitination is its role in directing most ubiquitinated proteins toward degradation via the 26S ubiquitin-proteasome system (UPS) [5,6,7]. The ubiquitination pathway comprises an enzymatic cascade involving three consecutive enzymes: E1 ubiquitin-activating enzyme (E1), E2 ubiquitin-conjugating enzyme (E2), and E3 ubiquitin ligases (E3) [6]. Among these, E3 ligases are the primary determinants of substrate specificity for ubiquitination. They are integral components of the ubiquitination pathway, generating protein-specific signals for the UPS by selectively recognizing target proteins [8,9]. Consequently, E3 ligases are diverse and abundant within cells. In plants, E3 ligases are pivotal not only for protein degradation but also for regulating various cellular and physiological processes such as phytohormone signaling, light responsiveness, and tolerance to biotic and abiotic stresses [10,11,12]. Various types of E3 ubiquitin ligases have been identified, including Really Interesting New Gene (RING) [13], Homology to E6-AP Carboxy Terminus (HECT) [14], U-box [15], cullin-RING [16], and Anaphase Promoting Complex type (APC) [17,18].

The documented significance of HECT ubiquitin ligases in plant growth, development, and abiotic stress responses highlights their vital role. This subclass of E3 ligases is characterized by a distinctive 350-amino acid HECT domain at the C-terminus [19,20]. In terms of their structure, HECT E3 ligases exhibit a format comprising a catalytic HECT domain located at the C-terminal (C-lobe) and a less conserved N-terminal region (N-lobe). The C-lobe hosts an essential cysteine (Cys) active site that is crucial for the formation of a transient thioester bond required for ligation, while the N-lobe collaborates with E2 to facilitate effective ubiquitin transfer. The variety of domains within the N-lobe is key in categorizing HECT E3 ligases into specialized subfamilies [19,20,21,22,23]. Furthermore, the N-terminal region in HECT E3s features substrate-binding domains, proficient in identifying specific sequences such as the PY motifs of substrates.

In plants, the HECT gene family in *Arabidopsis*, the smallest among E3 subfamilies, encompasses seven genes denoted as *UPL1-UPL7*. Research into the function of *Arabidopsis’s UPL3* and *UPL5* has revealed *UPL3*’s role in trichome development and *UPL5*’s association with leaf senescence [21,24,25]. Numerous HECT members have been recognized in various species, including 12 in *Zea mays* [26], 6 in *Oryza sativa* [14], 15 in *Glycine max* [27], 13 in *Malus domestica* [28], 7 in *Sorghum bicolor* [14], 14 in *Solanum lycopersicum* [29], and 7 in *Arabidopsis thaliana* [14,21], emphasizing the significant contribution of HECT E3 ligases. Bamboo, known for its rapid growth and high lignocellulose content, stands out as a significant non-timber forest resource worldwide. Hence, identifying and characterizing HECTs in bamboo can significantly contribute to research focused on bamboo growth and its resilience to abiotic stressors. However, there has been a notable gap in the analysis of the HECT E3 ligases gene family in *Phyllostachys edulis*.

In this study, we conducted a comprehensive genome-wide examination and characterization of the HECT E3 ubiquitin ligase family members in *P. edulis*. Our analysis included a detailed analysis that encompassed phylogenetic distribution, gene structure features, conserved domains and motifs, collinearity assessment, and cis-regulatory elements. Additionally, we explored the expression profiles of these genes across various tissues and developmental stages, along with their response to diverse abiotic stresses and hormone treatments, using data sourced from public databases. We identified key stress-responsive genes and analyzed their co-expression regulatory networks. Subsequently, we focused on the hub gene, *PeHECT1*, which exhibited significant expression induction in response to cold and drought stresses, for further functional analysis in plants. This study aims to provide substantial insights that will facilitate further investigation into the molecular mechanism underlying HECT involvement in plant growth and stress response in *P. edulis*.

## 2. Results

### 2.1. Genome-Wide Identification and Characterization of PeHECTs in P. edulis

The conserved HECT domain (PF00623) was identified in both the NCBI Conserved Domain Database (https://www.ncbi.nlm.nih.gov/Structure/cdd/wrpsb.cgi, accessed on 12 December 2023) and the PFAM database (http://pfam.xfam.org/, accessed on 13 December 2023), forming the foundation for the Hidden Mark Model (HMM) employed in the comprehensive genome-wide identification of HECT E3 ligases. A total of 16 HECT E3 ligases were identified within the *P. edulis* genome. Essential characteristics, including the length of coding sequences, amino acid length, molecular weights, isoelectric point, and subcellular localization of these HECT E3 ligases, were thoroughly examined and were detailed in Table 1. The coding sequences (CDS) of *PeHECTs* varied in size from 2502 to 11,172 bp, corresponding to amino acid lengths ranging from 730 to 3723 and molecular weights spanning from 93.94 to 407.94 kDa. The theoretical isoelectric points of PeHECT E3 ligases ranged from 4.9 to 8.69. The majority of PeHECT E3 ligases exhibited predicted subcellular localization, as shown in Table 1.

**Figure 1:**
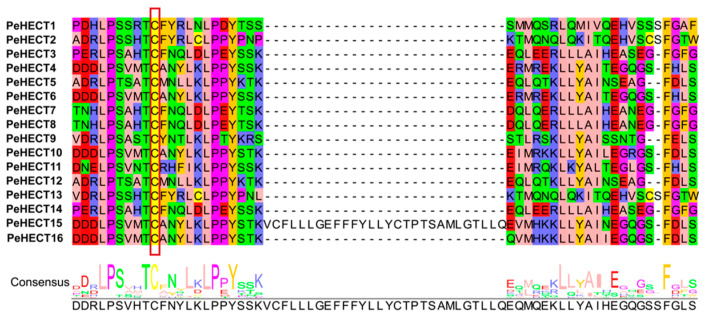
Alignment of the HECT domain surrounding the active-site cysteine residue in PeHECT E3 ligases. The red box indicates the conserved cysteine residue.

The comparative analysis of protein sequences surrounding the functionally conserved cysteine residue in the C-terminal HECT domain of PeHECT E3 ligases proteins revealed the preservation of the catalytic cysteine residue at the active center, along with other amino acid residues like leucine (L), proline (P), serine (S), threonine (T), and phenylalanine (F) that are highly conserved (Figure 1). These residues collectively play a vital role in governing the structural stability and catalytic efficacy of *P. edulis* HECT E3 ligases.

### 2.2. Phylogenetic Analysis of PeHECTs

**Figure 2:**
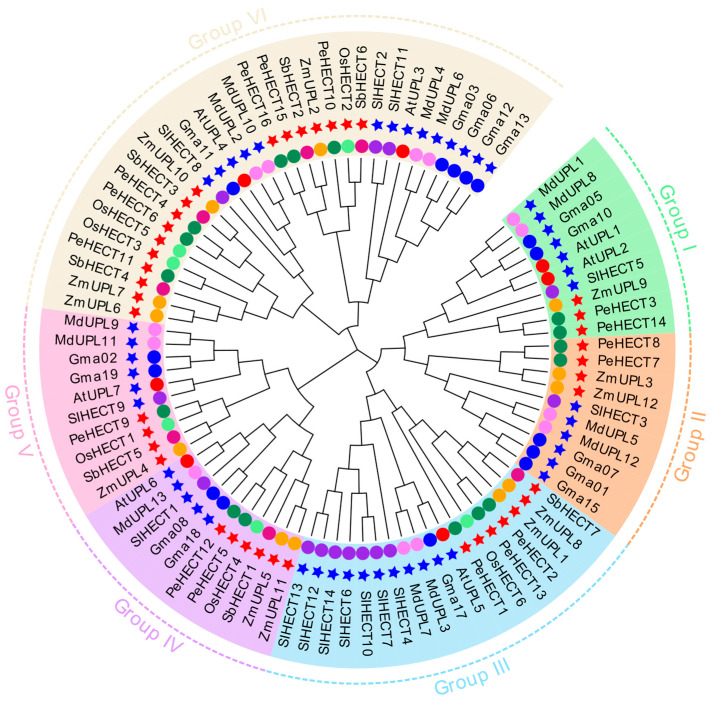
Phylogenetic tree of HECT E3 ligases in plants. A total of 90 HECT proteins were identified from *Phyllostachys edulis* (*Pe*), *Zea mays* (*Zm*), *Oryza sativa* (*Os*), *Glycine max* (*Gm*), *Malus domestica* (*Md*), *Sorghum bicolor* (*Sb*), *Solanum lycopersicum* (*Sl*), and *Arabidopsis thaliana* (*At*). The tree was divided into six groups, each indicated by a different color representing the background and labeled with individual HECT family names. Different colors in circles represent different species. Blue stars indicate dicotyledonous plants, while red stars indicate monocots.

A maximum likelihood phylogenetic analysis, with 1000 bootstrap replications, was conducted to elucidate the evolutionary relationships of HECT E3 proteins in *P. edulis*, alongside other extensively studied monocot and dicot plants. These species comprised 16 members in *Phyllostachys edulis*, 12 in *Zea mays*, 6 in *Oryza sativa*, 15 in *Glycine max*, 13 in *Malus domestica*, 7 in *Sorghum bicolor*, 14 in *Solanum lycopersicum*, and 7 in *Arabidopsis thaliana*. The analysis encompassed a dataset of 90 HECT E3 ligases across these species (Figure 2). Six distinct groups (Group I–VI) were identified through the phylogenetic analysis, consistent with research findings in soybeans. Notably, HECT family proteins of *Phyllostachys edulis*, *Oryza sativa*, *Zea mays*, and *Sorghum bicolor*, belonging to the *Gramineae* family, were clustered in the same subclade. Furthermore, it was observed that the majority of genes evolved subsequent to the divergence of monocots and dicots. Group VI, the largest cluster according to the phylogenetic tree, includes 6 PeHECT members, while Group V is the smallest, comprising only one PeHECT member. Similarly, *Oryza sativa*, *Zea mays*, *Sorghum bicolor* each possess a single member in the analysis.

### 2.3. Gene Structure and Conserved Protein Motifs of PeHECTs

**Figure 3:**
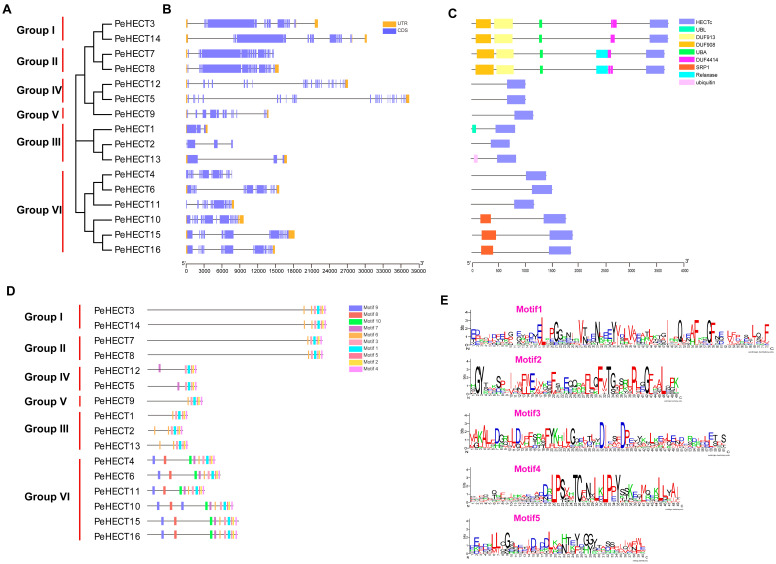
Gene structures, conserved motifs, and conserved domains of *P. edulis* HECT members. (**A**) Phylogenetic tree generated using MEGA11. (**B**) Exon/intron structure of putative *PeHECT* genes. Blue boxes indicate exons, while orange boxes represent 3′ or 5′ UTRs (untranslated regions). (**C**) Domain architectures of *P. edulis* HECT E3 ligases based on phylogenetic relationships, with each domain represented by a colored box. (**D**) Motif compositions of PeHECT E3 ligases, with differently colored boxes representing different motifs. (**E**) Sequence logo of conserved motifs one to five in the HECT domain.

The phylogenetic tree illustrating the expansion of PeHECT members in *P. edulis* was meticulously constructed using MEGA11.0.13. Within this phylogenetic representation, individual group members were distinctly clustered together (Figure 3A). The gene architecture of the PeHECT family was examined using genomic DNA sequences, as represented in Figure 3B. Within the PeHECT cluster, the number of exons ranged from 3 to 27, with a notable observation that 10 genes each contained 10 exons. The genomic DNA sequence lengths exhibited significant diversity, spanning from 3587 bp to 37,392 bp. Notably, most members within this gene subfamily displayed a similar structural organization, as illustrated by the sequence similarity observed between *PeHECT15* and *PeHECT16*.

The conserved domain analysis of PeHECTs was performed using the CD-search tool on the NCBI platform, identifying nine domains: the HECT domain (HECT), ubiquitin domain (UBQ), ubiquitin-like domain (UBL), ubiquitin-associated domain (UBA), ubiquitin-binding motif (UBM), domain of unknown function (DUF), armadillo (ARM) repeat, and relaxase domain. Apart from the HECT domain, the N-terminal regions of PeHECT E3 ligases were revealed to be crucial for facilitating interactions with diverse substrates (Figure 3C). Groups I and II included the DUF, UBA, and UBM domains, with the relaxase domain additionally present in Group II. PeHECT1 and PeHECT13, classified under Group III, each featured the UBL and UBQ domains, respectively. Moreover, PeHECT10, PeHECT15, and PeHECT16 each displayed an ARM domain.

Subsequently, the MEME software version 5.5.7 was utilized to examine the conserved motifs in the protein structure of PeHECTs, revealing a total of 10 conserved motifs within the PeHECT E3 ligases (Figure 3D). Particularly, motifs one to five were associated with the conserved HECT domain, as illustrated in Figure 3E, where the motif logo was displayed in Figure 3E. The consistent distribution and quantity of motifs among subfamily members suggest the preservation of functional characteristics within this protein group.

### 2.4. Chromosomal Location, Collinearity, and Evolution Analysis of PeHECTs

**Figure 4:**
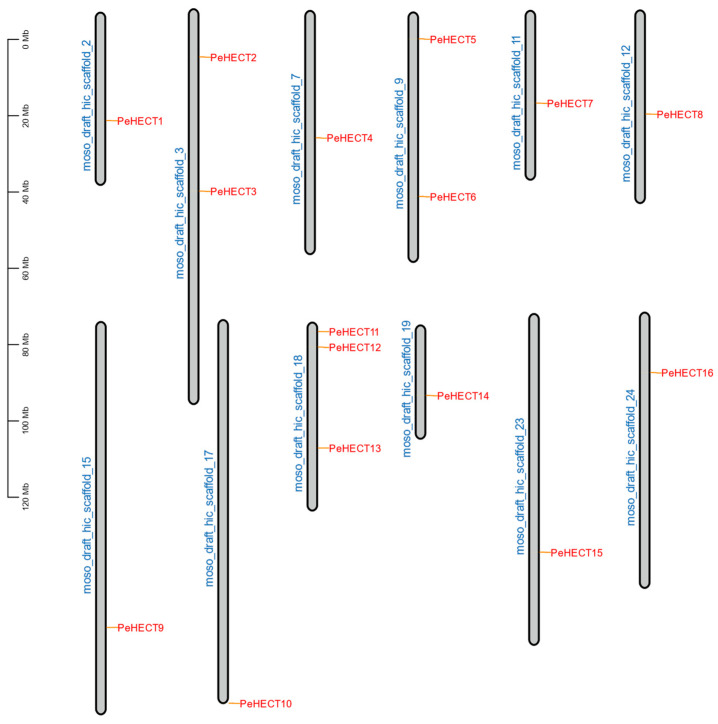
Scaffolds distribution of PeHECTs in *P. edulis*. The scale denotes the length of the *P. edulis* scaffolds.

To investigate the chromosomal distribution of PeHECTs in *P. edulis*, HECT genes were genetically mapped onto 24 chromosomes (scaffold 1–24). Within *P. edulis* genome,16 HECT genes were unevenly distributed across 12 of the 24 scaffolds (Figure 4). This irregular distribution of expanded genes on each scaffold could be attributed to the incomplete status of the genome and the limited genetic variation inherent in *P. edulis*. Specifically, three HECT genes were located on scaffold eighteen, while scaffolds three and nine each held two genes. On the other hand, scaffolds 2, 7, 11, 12, 15, 17, 19, 23, and 24 each contained a single gene.

**Figure 5:**
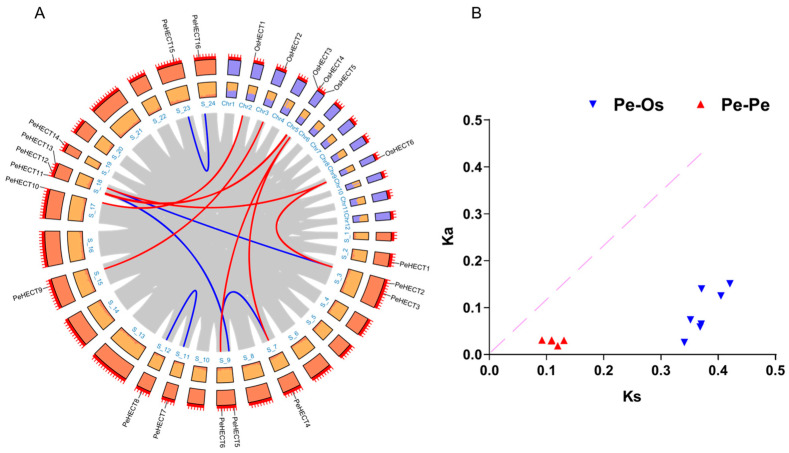
Synteny relationships and *Ka/Ks* ratios of gene pairs in the synteny regions of *PeHECTs* and *OsHECTs*. (**A**) Duplicated genes between *PeHECTs* and *OsHECTs* are indicated with red lines, while duplicated genes within *PeHECTs* are indicated with blue lines. (**B**) Analysis of *Ka/Ks* ratios for gene pairs of *PeHECTs-PeHECTs* and *PeHECTs-OsHECTs*.

Rice, a prominent model plant crop, demonstrates well-defined genomic functions. Both rice and *P. edulis* belong to the *Gramineae* plant family. To investigate gene duplication events of HECT genes in *P. edulis* and *Oryza sativa*, a collinearity analysis was conducted using MCscanX and Tbtools 2.0. Through meticulous comparison, seven potential orthologous gene pairs (*Pe-Os*) and five putative paralogous gene pairs (*Pe-Pe*) were identified, suggesting a shared ancestor among these genes (Figure 5A). However, it was observed that certain genes in *P. edulis* lacked corresponding synteny genes; for instance, *PeHECT1* and *PeHECT14* showed no synteny with other genes. This could be attributed to multiple chromosomal rearrangements, fusions, and obscured chromosomal syntenies resulting from selective gene losses within the *P. edulis* and *Oryza sativa* (*O. sativa*) genomes.

The evolutionary pressure on HECT gene pairs within direct homologous genes in bamboo and between corresponding homologous genes in bamboo and rice was investigated. A total of 12 homologous gene pairs were identified, comprising 5 homologous genes from bamboo and 7 from both bamboo and rice, involving 13 bamboo genes and 5 rice genes. Typically, a *Ka/Ks* ratio less than one indicates negative or purifying selection, equal to 1 denotes neutral selection, and over one suggests positive selection [30,31]. Negative selection pressure was observed in all HECT ortho/paralog homologous gene pairs, with significant purifying pressure at 0.076 in *OsHECT2-PeHECT9* and reduced purifying pressure at 0.377 in *OsHECT6-PeHECT13* (Figure 5B). The divergence time of HECT genes in *Phyllostachys* ranged from approximately 91.95 million years ago (*PeHECT7-PeHECT8*) to about 10.03 million years ago (*PeHECT13-PeHECT2*). The divergence between *P. edulis* and *O. sativa HECT* genes was estimated to be between 32.31 million years ago (*OsHECT6-PeHECT2*) and 26.19 million years ago (*OsHECT2-PeHECT9*). The earliest differentiation of *HECT* gene family members (*OsHECT6-PeHECT2*) in *O. sativa* and *P. edulis* occurred approximately 32.31 million years ago, while the divergence of *PeHECT7* and *PeHECT8* in *P. edulis* occurred around 91.95 million years ago. Previous studies indicated that bamboo underwent whole-genome duplication around 7–12 MYA, while the split between rice and *P. edulis* occurred 48.6 MYA [32]. These findings suggest that the genome duplication event of the *HECT* gene family in *Phyllostachys* predates its divergence from *O. sativa* and *P. edulis*.

### 2.5. Cis-Acting Regulatory Elements Identification in Promoter of PeHECTs

**Figure 6:**
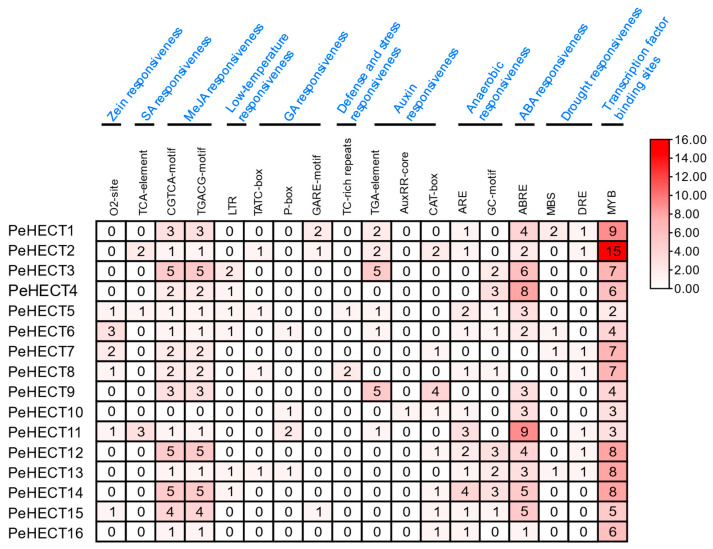
*Cis*-elements analysis associated with phytohormone and abiotic stress responsiveness in the promoter regions of PeHECTs. The heatmap demonstrates the number of cis-elements, with the higher number shown in red and the lower number shown in white.

To comprehensively understand the inherent biological functions and regulatory mechanisms, we examined the promoter regions of the 16 PeHECT genes by analyzing the upstream 2000 bp of transcriptional start site to discover cis-acting regulatory elements, utilizing the PlantCare database. Various responsive cis-elements were identified, critical in plant hormone metabolism and responses to abiotic stresses (Figure 6), including drought-responsive elements, abscisic acid (ABA) response elements, methyl jasmonic acid (MeJA) -responsive elements, gibberellin (GA) -responsive elements, auxin-responsive elements, salicylic acid (SA) -responsive elements, and others (Figure 6). The majority of HECT genes contain elements essential for hormonal responses, like auxin, GA, ABA, SA, and MeJA, indicating their significant roles in hormonal pathways. The most common cis-elements were counted, revealing ABA-responsive elements (ABRE motif) present in 14 PeHECT genes and MeJA-responsive elements (CGTCA-motif and TGACG-motif) present in 15 *PeHECT* genes, ARE motif in 12 genes, and AuxRE-core exclusively found in one gene. Noteworthy among the *PeHECT* promoters, 14, 4, 6, and 13 contained anaerobic, drought, low-temperature, defense, and stress responsiveness cis-elements, respectively (Figure 6). Additionally, most *PeHECT* promoters displayed binding sites for MYB transcription factors. In conclusion, these findings suggest that *HECT* genes likely participate in plant hormone signaling pathways and respond to various environmental stress facilitated by the regulation of multiple cis-elements.

### 2.6. Expression Pattern of PeHECTs in Different Tissues of P. edulis

**Figure 7:**
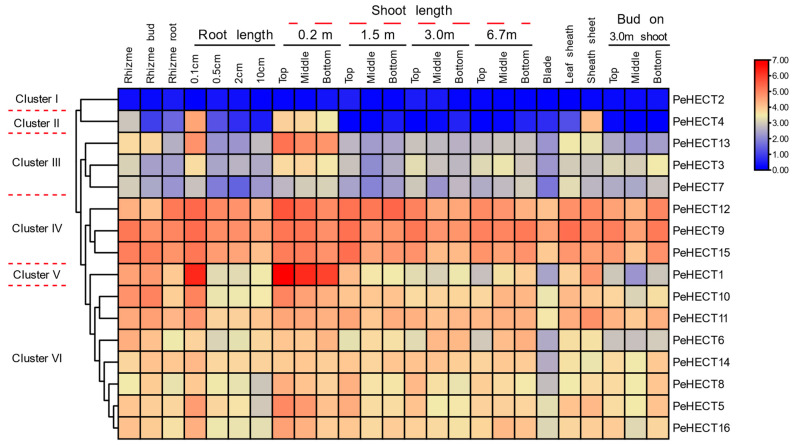
The expression patterns of PeHECTs in various tissues at developmental stages. The expression patterns were analyzed based on the RNA-seq data of *P. edulis*. The hierarchical clustering heatmap was plotted according to the FPKM values. The red color indicates high expression levels, and the blue color indicates low levels.

Transcriptome data from 26 different tissue samples representing various growth and developmental stages of *P. edulis* were employed to investigate the expression profiles of PeHECTs [33]. Genes were classified based on their specific tissue expression patterns observed in the data (Figure 7). Notably, *PeHECT2* consistently exhibited a low expression level across all tissues, while *PeHECT4* showed a distinct tissue-specific expression in the 0.1 cm length root, 0.2 m length shoot, and sheath sheet, contrasting with minimal expression in other tissues (Figure 7). Conversely, *PeHECT1* displayed a marked tissue-specific expression pattern, with its peak expression in the 0.1 cm length root and the 0.2 m length shoot, indicating a potential involvement in cell division. Similarly, *PeHECT13* showed a similar expression profile to *PeHECT1* but with lower expression levels (Figure 7). On the other hand, *PeHECT9*, *12*, and *15* demonstrated elevated expression levels across all tissues. The remaining genes did not exhibit a clear expression pattern. Collectively, these diverse expression profiles suggest varied functional implications for the respective PeHECT genes.

### 2.7. PeHECTs Response to Various Hormones and Stresses

**Figure 8:**
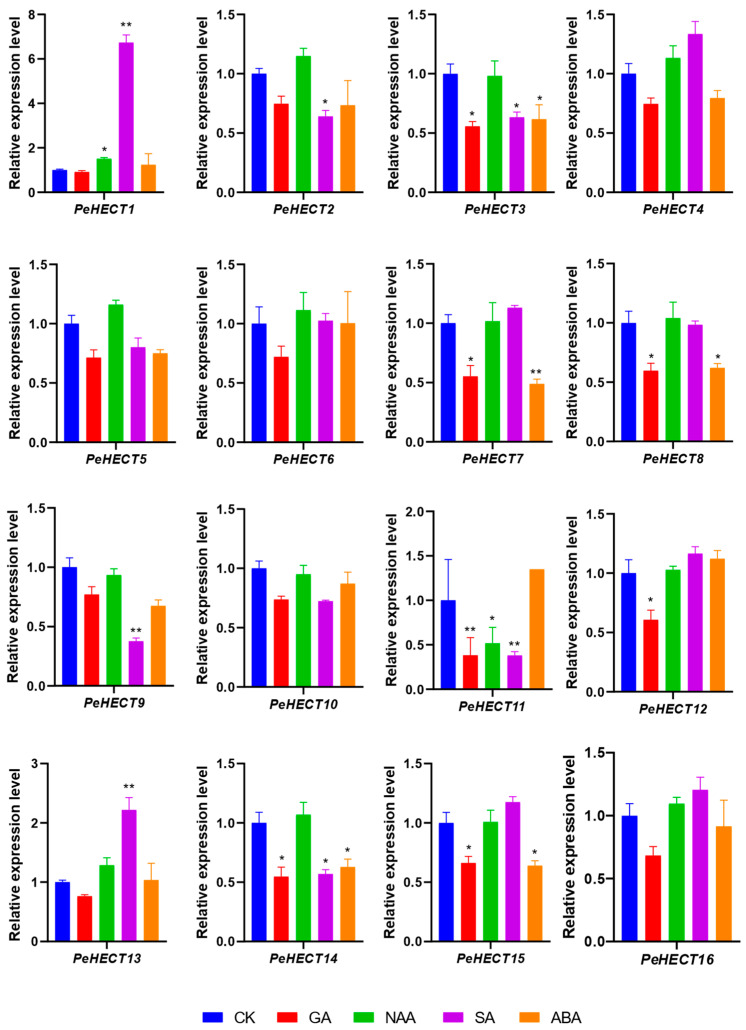
Expression patterns of PeHECT genes in response to different hormones. The relative expression levels of PeHECT genes were analyzed using RNA-seq data from *P. edulis* seedlings treated with CK, GA, NAA, SA, ABA. The expression of PeHECTs in seedlings treated with tap water was used as the reference. Data are presented as mean ± standard deviation. Student’s *t*-test was used to generate the *p* value; * *p* < 0.05, ** *p* < 0.01.

The availability of multiple transcriptome databases has enabled a thorough examination of gene expression patterns in PeHECT genes. The mRNA expression profiles of PeHECTs in responding to various abiotic stresses and hormones were investigated. In this study, a 1.5-fold difference in gene expression between treated and untreated controls (without being treated) at each treatment time point was classified as a significant difference. Under GA treatment, seven PeHECT genes manifested downregulation, while nine genes showed no significant change (Figure 8). Conversely, following naphthaleneacetic acid (NAA) treatment, only *PeHECT1* was upregulated by 1.5 times, and *PeHECT11* was downregulated. Upon SA treatment, two PeHECT genes were upregulated, and five genes were downregulated (Figure 8). Notably, *PeHECT1* showed a significant upregulation by 6.47 times compared to the control sample (Figure 8). Meanwhile, five PeHECT genes were significantly downregulated under ABA treatment. These differential expression patterns suggest a potential involvement of these genes in various biological processes in response to abiotic environmental stresses, such as dehydration and cold.

**Figure 9:**
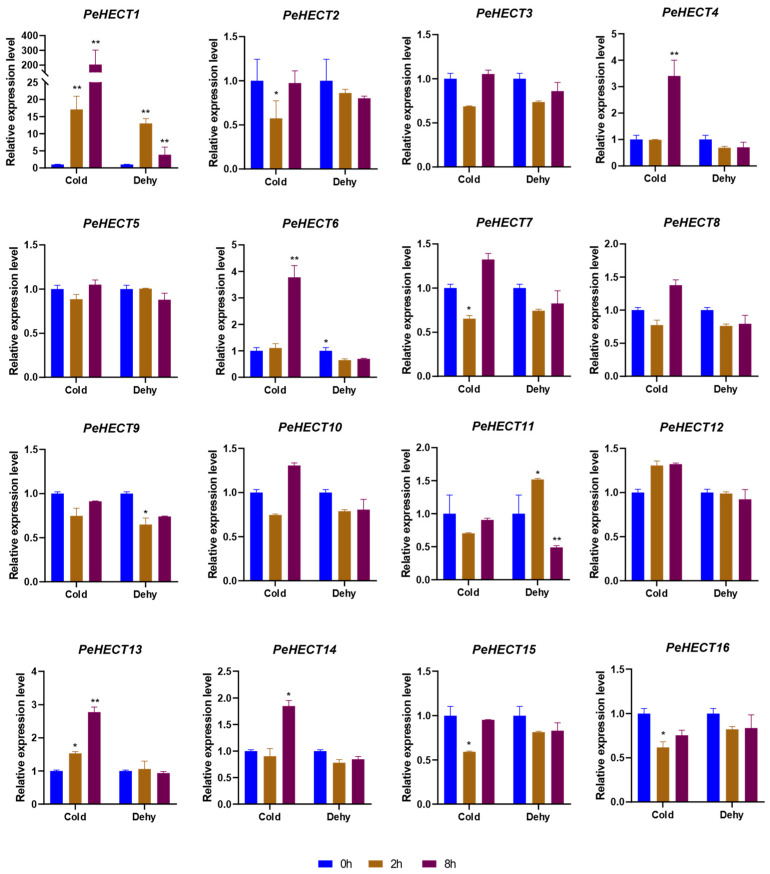
Expression levels of PeHECTs in response to dehydration and cold stress. The relative expression levels of PeHECT genes were analyzed using RNA-seq data from *P. edulis* seedlings treated with dehydration (dehy) stress and cold stress for 0 h, 2 h, and 8 h. Data are presented as mean ± standard deviation. Student’s *t*-test was used to generate the *p* value; * *p* < 0.05, ** *p* < 0.01.

While subjected to cold stress, five PeHECTs displayed upregulation, while four showed downregulation. Notably, the expression levels of *PeHECT1* and *PeHECT13* consistently increased over the 2 h and 8 h cold treatment (Figure 9). Under dehydration stress, one gene displayed upregulation, and two genes exhibited downregulation. Interestingly, one gene demonstrated upregulation at 2 h but downregulation at 8 h under dehydration stress (Figure 9). Significantly, during cold stress, *PeHECT1* showed a remarkable 17-fold and 203-fold increase compared to the control, while under dehydration stress, it exhibited an 8-fold increase at 2 h and a 3.87-fold increase at 8 h (Figure 9). These findings suggest that these genes likely play diverse roles in response to different abiotic stresses.

### 2.8. Co-Expression Analysis of PeHECTs

**Figure 10:**
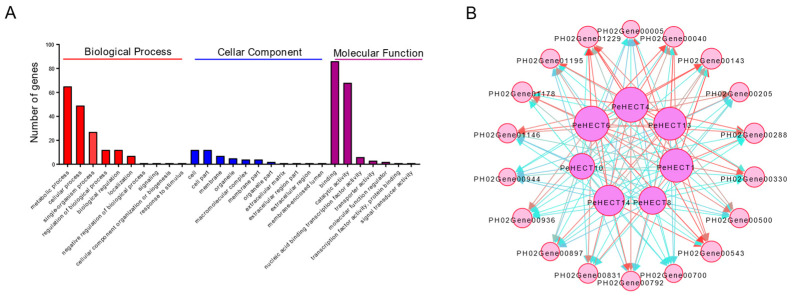
Co-expression network analysis of the PeHECT genes. (**A**) Gene enrichment analysis in the blue modules. (**B**) Hub genes co-expressed with PeHECTs in the blue modules.

To build a co-expression regulatory network of PeHECT genes, we performed a weighted gene co-expression network analysis (WGCNA) using [34,35] transcriptional profiling data from *P. edulis* seedlings exposed to cold and dehydration stress (Supplementary Figure S1A). We identified 17 distinct modules among the 29,981 nodes analyzed (Supplementary Figure S1B). We then determined the module to which the PeHECTs were assigned. Our analysis revealed that the blue module encompassed nine PeHECTs, specifically *PeHECT1*, *PeHECT4*, *PeHECT5*, *PeHECT6*, *PeHECT7*, *PeHECT8*, *PeHECT10*, *PeHECT13*, and *PeHECT14*. These genes within the blue module exhibited increased expression levels following an 8 h cold treatment. Subsequently, we identified genes co-expressed with these nine PeHECT genes in the blue module and established a co-expression network among them (Supplementary Figure S2). An examination of each Gene Ontology (GO) term associated with these neighboring genes revealed their involvement in metabolic process, cellular process, single-organism process, biological regulation, cell part, biological regulation, binding process, and catalytic activity (Figure 10A).

Additionally, we investigated the hub genes within this co-expressed network, which comprised seven PeHECT genes (Figure 10B). These hub genes encode proteins such as VQ motif protein, RTF2 RING-Finger protein, UBA/TS-N domain protein, 26S proteasome subunit, and G-protein alpha subunit (Table 2). These proteins may potentially play crucial roles in plant growth, development, and resistance to stress.

### 2.9. Overexpression of PeHECT1 Enhanced the Expression of Stress-Related Genes in Tobacco

**Figure 11:**
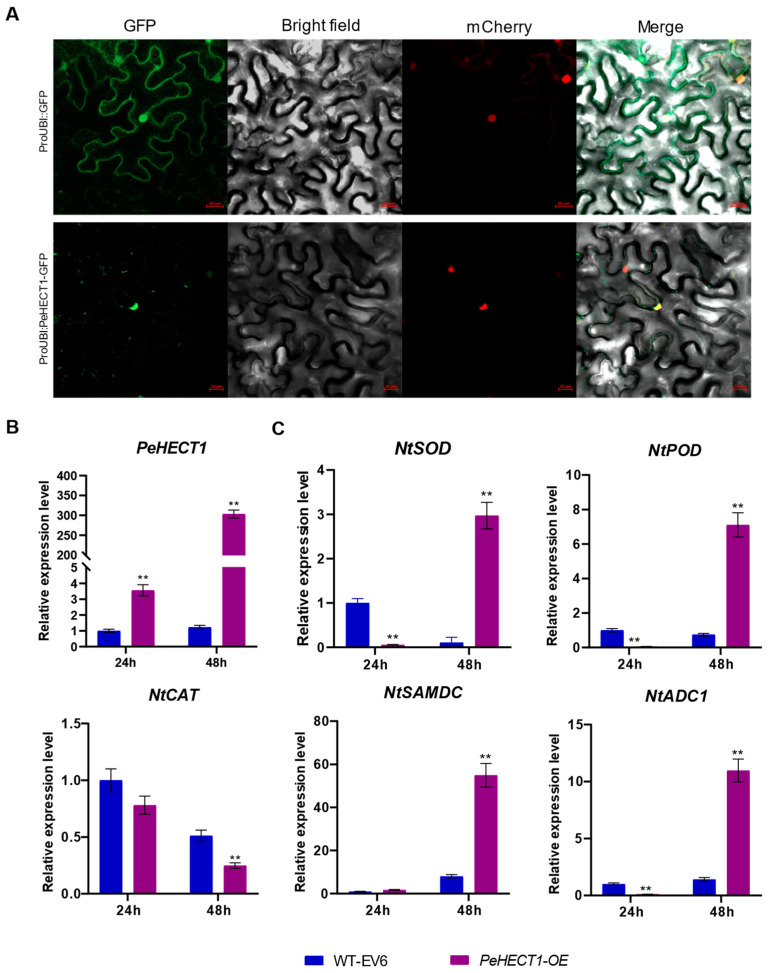
Subcellular localization of *PeHECT1* and its potential functional validation in tobacco. (**A**) Subcellular localization of *PeHECT1*. Scale bar = 20 μm. (**B**) Overexpression of *PeHECT1* in tobacco leaves. (**C**) Analysis of relative expression of stress-related genes in tobacco. Data are presented as mean ± standard deviation. Student’s *t*-test was used to generate the *p* value; ** *p* < 0.01.

To elucidate the function of PeHECTs, *PeHECT1* was chosen due to its expression being predominantly linked to cell division and its significant induction under cold and dehydration abiotic stresses. We generated a *PeHECT1*-GFP fusion construct with a green fluorescent protein to determine the subcellular localization of *PeHECT1*. The fluorescent signal was mainly detected in the nucleus (Figure 11A), consistent with the predicted subcellular protein localization.

Subsequently, we utilized the overexpression vector of *ProUBI*::*PeHECT1*-GFP fusion for transient transformation into tobacco leaf epidermal cells. Samples were collected at 24 h and 48 h post injection. Following RNA extraction, the gene expression of *PeHECT1* and tobacco stress-related genes were validated. The data revealed that the transient transfection with *PeHECT1* led to a significant 3.56-fold increase at 24 h, reaching a 303-fold increase at 48 h (Figure 11B). Relative expression levels of tobacco stress-related genes involved in ROS detoxification (*NtPOD*, *NtSOD*, *NtCAT*) and polyamine biosynthesis (*NtADC1* and *NtSAMDC*) at 24 h and 48 h post-transformation were evaluated (Figure 11C). The analysis revealed that the expression levels of *NtPOD*, *NtSOD*, and *NtADC1* were not induced at 24 h but rather downregulated, whereas they exhibited significant upregulation at 48 h post-injection. Specifically, at 48 h, the expression of *NtPOD*, *NtSOD*, and *NtADC1* was 9.51, 28.15, and 7.81 times higher, respectively, compared to the empty vector injection at 48 h. *NtCAT* did not display alterations at 24 h post-injection but showed a significant reduction at 48 h. While *NtSAMDC* revealed no change at 24 h post-injection, it increased by 6.82-fold at 48 h. These results indicated that overexpression of *PeHECT1* in tobacco may enhance stress resistance by modulating the expression of ROS and stress-responsive genes.

### 2.10. PeHECT1 Was Regulated by Transcription Factor PeERF3

**Figure 12:**
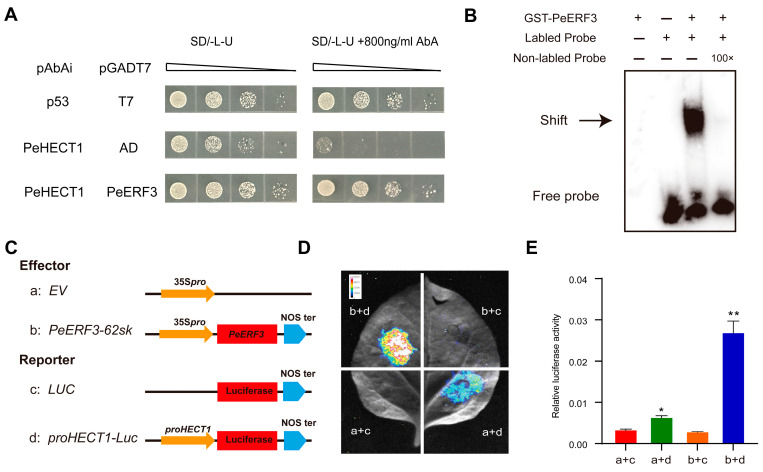
PeERF3 binds to the promoter of *PeHECT1* and activates its transcription activity. (**A**) Y1H assay of PeERF3 binding the promoter of *PeHECT1*. (**B**) EMSA assay of PeERF3 binding DNA probes. (**C**) Schematic diagrams of the effector (PeERF3) and reporter (proPeHECT1) constructs used in the dual-luciferase reporter assay. (**D**) Transcriptional activities of PeERF3 on *PeHECT1* promoter in tobacco epidermal cells. (**E**) Relative luciferase activity of PeERF3 on *PeHECT1* promoter. Data are presented as mean ± standard deviation. Student’s *t*-test was used to generate the *p* value; * *p* < 0.05, ** *p* < 0.01.

Based on the cis-acting regulatory elements identification in the promoter of *PeHECTs*, we observed the presence of dehydration-responsive element (DRE) in the promoter of *PeHECT1*. We utilized the PlantRegMap website (https://plantregmap.gao-lab.org/index.php, accessed on 20 January 2024) to predict the regulatory transcription factors. The results indicated that *PH02Gene28636*, which encodes an AP2/ERF family protein homologous to OsERF3, its overexpression enhanced the rice tolerance to drought, high salinity, and low-temperature stresses, could potentially bind to the promoter of *PeHECT1*. We employed the yeast one hybrid (Y1H) system to determine whether PeERF3 regulates *PeHECT1*. As expected, PeERF3 was directly bound to the promoter of *PeHECT1* (Figure 12A). Furthermore, the electrophoresis mobility shift assay (EMSA) indicated that the PeERF3 protein retarded the shift speed of the probe containing DRE motifs from the promoter of *PeHECT1*, which confirms the binding relationship between the PeERF3 protein and the DRE element (Figure 12B). Finally, a transient transcriptional activity assay with a LUC reporter was performed to analyze the transcription effect of PeERF3 on *PeHECT1*, and the luciferase transient transcriptional activity assay results revealed that PeERF3 activated the transcriptions of *proPeHECT1*::LUC, indicating that PeERF3 directly regulated the expression of *PeHECT1* (Figure 12C–E). These results indicated that *PeHECT1* was the downstream and regulated by the transcription factor of PeERF3.

### 2.11. Evolutionary Relationship of PeHECT1 Among Bamboo Species

**Figure 13:**
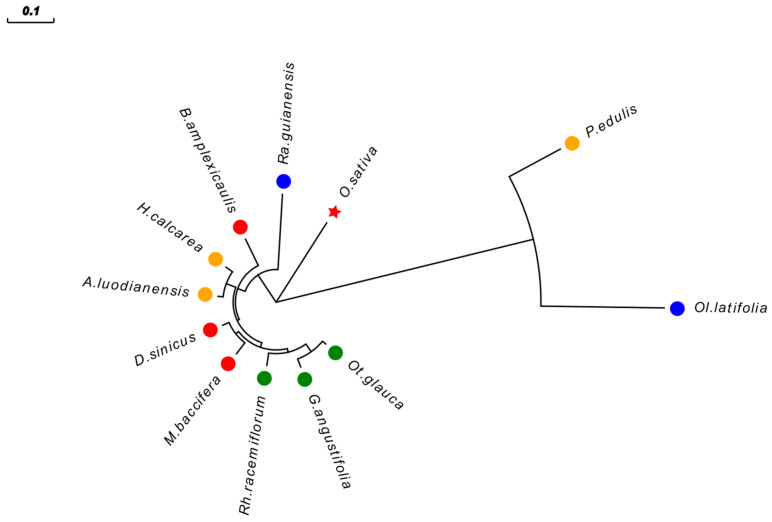
Evolutionary relationship of PeHECT1 among different bamboo species and rice. As shown in the figure: *Phyllostachys edulis* (*P*. *edulis*), *Oryza sativa* (*O*. *sativa*), *Raddia guianensis* (*Ra*. *guianensis*), *Dendrocalamus sinicus* (*D*. *sinicus*), *Bonia amplexicaulis* (*B*. *amplexicaulis*), *Hsuehochloa calcarea* (*H*. *calcarea*), *Ampelocalamus luodianensis* (*A*. *luodianensis*), *Melocanna baccifera* (*M*. *baccifera*), *Rhipidocladum racemiflorum* (*Rh*. *racemiflorum*), *Guadua angustifolia* (*G*. *angustifolia*), *Otatea glauca* (*Ot*. *glauca*), and *Olyra latifolia* (*Ol*. *latifolia*). Red circle indicates the paleotropical woody bamboo, green circle indicates neotropical woody bamboo, yellow circle indicates temperate woody bamboo, blue circle indicates herbaceous bamboo, and red star indicates rice.

To date, 11 high-quality genome sequences of bamboo species have been publicly released, providing significant data support [36] for the study of the systematic evolution and functional analysis of bamboo plants. By utilizing Bamboo Base, a genomic and taxonomic information platform accessible at https://bamboo.genobank.org/, accessed on 10 April 2024, homologous sequences of PeHECT1 were identified in multiple bamboo species, with these protein sequences found in 11 bamboo species but absent in *D. latiflorus* (Figure 13). In a comparative analysis of the evolutionary relationships among 11 bamboo species and rice, it was found that the PeHECT1 in *P. edulis* exhibited a close association with the diploid herbaceous bamboo *Ol. latifolia*, followed by the divergence of rice and bamboo. Furthermore, the presence of *PeHECT1* in *B. amplexicaulis* of neotropical woody bamboo preceded its presence in other temperate and tropical woody bamboo species.

## 3. Discussion

In this study, 16 HECT genes were identified in *P. edulis*, which were mapped across 12 distinct scaffolds. Comparative analysis of protein sequences revealed the preservation of the catalytic cysteine residue at the active center in the C-terminal HECT domain of PeHECTs. Phylogenetic analysis revealed that HECT gene family in *P. edulis* exhibits relative conservation when compared to rice and maize; however, it has undergone significant expansion, resulting in an increased number of members. Notably, PeHECT members predominantly localize to the nucleus, cell membrane, and chloroplast. Phylogenetic analysis revealed a close evolutionary relationship between *P. edulis HECT* members and those of other gramineous crops, with monocot species forming a distinct cluster. The distinctive domains of the PeHECTs could be attributed to variations in conserved motifs throughout evolution. Gene duplication events are crucial for enabling organisms to adapt to varying environmental conditions during growth and development, representing a significant evolutionary mechanism for the rapid expansion and diversification of gene families. We analyzed the *Ka/Ks* ratio for five paralogous genes (*Pe-Pe*) and seven orthologous genes (*Pe-Os*) within the HECT gene family, revealing negative selection pressure across all HECT ortho/paralog homologous gene pairs. Our study discovered that gene duplication events occurred at approximately 91.95 MYA in *P. edulis*, with the divergence time for orthologous genes (*Pe-Os*) estimated at around 32.32 MYA. According to [32], the divergence time between *P. edulis* and rice occurred around 48.6 MYA, suggesting that the genome replication event in *Phyllostachys* predates its divergence from rice and *P. edulis*.

An analysis of gene expression patterns across various tissues and developmental stages revealed that *PeHECT12*, *PeHECT9*, and *PeHECT15* exhibited high expression in nearly all tissue types and developmental stages. Conversely, *PeHECT1*, *PeHECT4*, and *PeHECT13* demonstrated significant overexpression in 0.1 cm roots and 0.2 m shoots. This observation is consistent with previous research on other plant species [24,27], indicating that individual members of the HECT gene family perform tissue-specific functions throughout their lifecycle. In soybeans, most HECT genes showed elevated expression levels in flowers and roots, implying a potential role in the ubiquitination-mediated degradation of genes involved in flowering during the soybean flowering stage [27]. In *Arabidopsis thaliana*, the regulatory protein *AtUPL5* governs leaf senescence by targeting the degradation of WRKY53, a transcription factor implicated in leaf senescence promotion [24]. Our study identified *PeHECT1*, *PeHECT2*, and *PeHECT13* as the *P. edulis* genes orthologous to *AtUPL5*. Both *PeHECT1* and *PeHECT13* were expressed in tissues undergoing cell division, a process induced by NAA and associated with auxin-responsive elements in their promoter regions. Expression variations among paralogous genes in the same group suggest that HECT genes in *P. edulis* could share functional similarities with their *Arabidopsis thaliana* orthologs while potentially evolving distinct functions.

Plant growth and development can be impeded by various environmental stressors, highlighting the challenge of maintaining a balance between plant growth and stress resistance [37]. In *P. edulis*, numerous PeHECT genes demonstrated increased expression levels in response to cold and dehydration stresses, which are common challenges faced by bamboo species due to climate change. Within the HECT genes of *P. edulis*, *PeHECT1* stood out as the only gene receptive to both cold and dehydration, potentially due to its expression in specific tissues like the root and young shoots. Therefore, it can be inferred that the elevated expression of *PeHECT1* in roots and young shoots may contribute to the degradation of genes related to cold and drought-resistant proteins.

In response to adverse stress conditions, superoxide dismutase (SOD) serves as a primary defense enzyme against ROS by catalyzing the dismutation of H_2_O_2_ to protect cells from oxidative damage induced by environmental challenges. Catalase (CAT) and peroxidase (POD) cooperate to eliminate the produced hydrogen peroxide [38]. Enhanced expression of *PeHECT1* in tobacco has been observed to significantly increase the expression of tobacco genes *NtPOD* and *NtSOD*. This upregulation indicates the potential of *PeHECT1* to reduce H_2_O_2_ and O_2_^•−^ levels in leaves under abiotic stress, maintaining cellular redox balance and protecting cells from oxidative stress damage. However, there was no significant elevation in *NtCAT* expression, suggesting its role in eliminating peroxides other than hydrogen peroxide, thereby enhancing resilience to harsh conditions. Another pair of genes involved in the synthesis of polyamines, *NtADC1* and *NtSAMDC*, are implicated in adaptive responses to diverse environmental stresses [38,39,40]. Polyamines are crucial for maintaining ROS homeostasis [41]. Transient heterologous expression of *PeHECT1* resulted in an increase in the expression levels of *NtADC* and *NtSAMDC*, underlying the key role of *NtADC* and *NtSAMDC* in decreasing ROS accumulation in plants overexpressing *PeHECT1* plants. HECT may participate in regulating some proteins in the ROS signaling pathway to avoid excessive accumulation of ROS by interacting with and promoting the ubiquitination and degradation of these proteins involved in the clearance of ROS and free radicals, thus enhancing plant resistance.

The transcription activity of genes was regulated by upstream transcription factors binding to the specific sequences in promoters, which may serve as important regulators in abiotic stress processes. We obtained that PeERF3 was the upstream transcription-activated factor of *PeHECT1*, which bound to the DRE element in the promoter of *PeHECT1*. In rice, the homologous gene OsERF3/AP37 confers drought and high salt tolerance through the activation of stress tolerance genes [42,43]. In *P. edulis*, we found that PeERF3 could activate the transcription activity of *PeHECT1*, suggesting that the ERF transcription factors may also regulate abiotic stress tolerance using the ubiquitination process.

Prior research posits that the ancestor of bamboo was a diploid herbaceous, which subsequently evolved into tetraploid new tropical woody bamboo, tetraploid temperate woody bamboo, and hexaploid old tropical woody bamboo [44]. Comparative evolutionary analysis involving 11 bamboo species and rice revealed that the *PeHECT1* gene in *Phyllostachys edulis* shares a common ancestry with the diploid herbaceous bamboo *Olyra latifolia*, with subsequent close relationships observed with rice and herbaceous bamboo, suggesting an ancient origin of the *PeHECT1* gene in *Phyllostachys edulis*. However, the absence of *PeHECT1* in *Dendrocalamus latiflorus* could be attributed to the *PeHECT1* sequence’s association with the D subgenome. Even though rice and bamboo plants are currently classified in distinct subfamilies, earlier taxonomic studies positioned rice under *Bambusoideae*. Furthermore, a clear genomic collinearity exists between rice and bamboo plants. Our research uncovers the collinearity between orthologous genes in rice and *P. edulis*, with *PeHECT1* in *P. edulis* demonstrating a closer alignment with rice relative to other woody bamboo species. The emergence of the *PeHECT1* gene in *Bonia amplexicaulis*, a neotropical woody bamboo, predates that in other temperate and tropical woody bamboo. It has been suggested that ancient climatic and geological events were key catalysts for bamboo evolution. Moreover, the evolutionary relationships of *PeHECT1* within woody bamboos correspond to the evolutionary processes of woody bamboos. The significant elevation in *PeHECT1* expression, approximately 203-fold higher than the control, supports the proposition that *PeHECT1* in *P. edulis* represents a vital cold-resistant genotype preserved from ancient climates. The *PeHECT1* genotype in *P. edulis* could potentially be an ancient allele in bamboo.

## 4. Materials and Methods

### 4.1. Genome-Wide Identification of PeHECTs in P.edulis

A genome-wide identification of *HECT* members in *P. edulis* was facilitated by obtaining genomic, cDNA, and protein sequences of *P. edulis* from the BambooGDB database (http://www.bamboogdb.org/ssr, accessed on 10 January 2024). These sequences were analyzed using the Hidden Markov Model (HMM) with Pfam number PF00632, known for its conserved HECT domain. Additionally, sequences from rice *HECTs* were used to facilitate alignment with the protein sequences in *P. edulis*. The 16 *P. edulis* HECT members identified in *P. edulis* were designated as *PeHECT1* to *PeHECT16.* Any sequences that could not be mapped to any of the 24 chromosomes were excluded from the study. The Plant-mPLoc (http://www.csbio.sjtu.edu.cn/bioinf/plant-multi/#, accessed on 27 January 2024) was utilized to predict the subcellular localization of HECT E3 ligases (Table 1).

### 4.2. Phylogenetic Analysis

To compare HECT E3 ligases in *P. edulis* with those in well-studied monocot and dicot plant species (*Zea mays*, *Oryza sativa*, *Glycine max*, *Malus domestica*, *Sorghum bicolor*, *Solanum lycopersicum*, *Arabidopsis thaliana*), their respective protein sequences were aligned using the ClustalW 2.1 tool. Following this, phylogenetic analysis was conducted using MEGA11.0.13 with the maximum likelihood method with 1000 bootstrap replicates. The resulting phylogenetic tree was then visualized and enhanced visually using Evolview (https://evolgenius.info//evolview-v2/#login, accessed on 25 February 2024).

Homologous protein sequences of *PeHECT1* in *P. edulis* were retrieved from the website (http://www.genobank.org/bamboo, accessed on 10 April 2024) for 11 distinct bamboo species and rice, referencing previously published data [45]. The evolutionary relationships of *PeHECT1* across these bamboo species were established using the protein alignment and phylogenetic tree construction methods mentioned earlier.

### 4.3. Analysis of Gene Structure, Motif Identification, Conserved Domains, and Physical Location

To analyze the *HECT* gene structure in *P. edulis*, Tbtools 2.0 software was utilized to generate exon-intron maps based on genomic and coding sequences. The conserved domain analysis of *PeHECTs* was conducted using the CD-search tool available at NCBI. Conserved motifs were identified using the MEME tool (http://meme-suite.org/tools/meme, accessed on 2 March 2024) and visualized using TBtools 2.0 [46]. Furthermore, Tbtools 2.0 was utilized to illustrate the physical location on the chromosome.

The 2000 bp genome sequences upstream of the transcriptional start site were analyzed using the PlantCARE database (http://bioinformatics.psb.ugent.be/webtools/plantcare/html/, accessed on 25 March 2024) to predict cis-acting regulatory elements, encompassing stress responsiveness, hormone responsiveness, and transcription factor binding sites. The number of these elements was counted and displayed in a heatmap for visualization.

### 4.4. Synteny and Gene Duplication Analysis

To explore synteny within the genome, MCScanX in Tbtools 2.0 was employed to identify synteny regions within *P. edulis* and between *P. edulis* and rice. The relationships among genes were visually represented using the advanced circos in Tbtools 2.0 [46]. Collinear gene pairs were identified to trace the evolutionary expansion of genes, and Ks values and *Ka/Ks* ratios were calculated for these gene pairs. Ks values were utilized to estimate the timing of large-scale duplication events via the formula T = *Ks/2λ*, with λ was set at 6.5 × 10^9^, based on previous research regarding synonymous substitution rates in *P. edulis* and rice [32].

### 4.5. Expression Analysis of HECT Genes in P. edulis Based on RNA-Seq Data

To examine the expression patterns of PeHECT genes across various tissues and developmental stages in *P. edulis*, RNA-seq data from 26 samples were obtained from the NCBI Short Read Archive (SRA) with accession numbers ranging from SRX2408703 to SRX2408728 and SRR13201212 to SRR13201244. This analysis focused on evaluating gene expression in various plant parts such as rhizomes, roots, buds, leaves, sheaths, and shoots [33]. Additionally, to evaluate the responsiveness of PeHECTs to different hormone treatments and abiotic stresses, transcriptome data from *P. edulis* were utilized. Specifically, *P. edulis* seeds were germinated and grown in a soil mixture containing peat moss and vermiculite (volume ratio 2:1) in a greenhouse at 23 °C with a 16 h light/8 h dark photoperiod. Seedlings of about 4-week-old were used for hormone treatments. The analyzed hormone treatments included 100 μM of gibberellin (GA) (accession numbers SRR6131113-SRR6131118) and 5 μM of naphthaleneacetic acid (NAA) (accession numbers SRR5710697-SRR5710702) [34], 1 mM of salicylic acid (SA), and 1 μM of abscisic acid (ABA) (Gene Expression Omnibus accession number GSE169067) [47]. After *P. edulis* was treated with GA, NAA, SA, and ABA for 3 h, the samples were collected and immediately frozen in liquid nitrogen and then stored at −80 °C. In addition, 2 h and 8 h cold and dehydration abiotic stress treatments were also carried out (accession numbers SRR4450542-SRR4450551) [35]. Seedlings of about 4-week-old were used for abiotic stress treatments. For drought treatment, the whole seedlings were directly exposed to the air. For cold treatment, 4-week-old seedlings were placed in an environment of −2 °C. All samples were collected after 0, 2, and 8 h of treatment, immediately frozen in liquid nitrogen, and then stored at −80 °C.

### 4.6. Construction of Co-Expression Network

Transcriptome data from the leaves of 2-year-old *P. edulis* branches subjected to cold and dehydration treatments for 2 h and 8 h were utilized for weighted gene co-expression network analysis (WGCNA) using the analysis tools available at https://cloud.metware.cn/#/user/login, accessed on 7 April 2024 [35]. A total of 29,981 genes were retained for constructing the co-expression network using default settings with minor adjustments: a soft thresholding power of 18, a minimum module size of 50, a merge Cutree Height of 0.25, and a KME cutoff of 0.8. The resulting networks were visualized with Cytoscape v3.10.0, and hub genes within each module were identified using the cytoHubba plugin in Cytoscape v3.10.0.

### 4.7. Subcellular Localization of PeHECT1

To determine the subcellular localization of *PeHECT1*, the full-length cDNA of *PeHECT1* amplified using primer listed in Supplementary Table S1 was cloned into *pCAMBIA1300-Ubi::GFP-3×FLAG* vector. The resulting construct *PeHECT1*-GFP was then transformed into tobacco epidermal cells. Pick a single colony of the transformed GV3101 (containing the *PeHECT1*-GFP vector) from the plate and inoculate it into a small volume (5–10 mL) of liquid LB medium with the appropriate antibiotics. Incubate the culture overnight at 28 °C with shaking (around 200–250 rpm). The green fluorescent protein (GFP) signal was observed using laser scanning confocal microscopy (Zeiss LSM 700, Oberkochen, Germany).

### 4.8. RNA Extraction and qRT-PCR Analysis

Total RNA was extracted from tobacco leaves and transformed with the *PeHECT1* overexpression using the RNAprep Pure kit (Tiangen, DP432, Beijing, China). The PrimeScript 1st Strand cDNA Synthesis kit (Takara, No.6110A, Dalian, China) was employed to reverse transcribed to cDNA. The resulting cDNA was employed to assess gene expression levels, utilizing specific primers for stress-related genes and the *UBIQUTIN* gene as an internal control [48]. The primers for quantitative real-time PCR (qRT-PCR) were designed using the Primer 5.0 software (Supplementary Table S1). The reaction mixture included 5 μL of 2×Q3 SYBR qPCR Master Mix-Universal (ToloBio, #22204, Shanghai, China), 0.5 μL of each forward and reverse primer, 1 μL of cDNA template, and 3 μL of RNase-free water. The qRT-PCR was performed using QuantStudio^TM^7 Flex Real-time PCR instrument (Applied Biosystems, Carlsbad, CA, USA).

### 4.9. Yeast One Hybrid Assay

The 2000 bp promoter region upstream from ATG of *PeHECT1* was cloned into the pAbAi vector, respectively, and the full length of PeERF3 was cloned into pGADT7. The promoter fuse plasmid and ERF3-PGADT7 fuse constructs transformed into Y1H yeast competent cells and co-expressed in yeast were plated on SD/-Trp/-Ura plates containing different concentrations of AbA. The pAbAi-PeHECT1 promoter with empty PGADT7 was used as a control.

### 4.10. EMSA Assay

The CDS of PeERF3 was constructed to pGEX-4T1 to obtain a fusion protein with GST tag, and the promoter of *PeHECT1* containing DRE core region was used as a probe was 5′-end labeled using Biotin and combined with double-stranded using PCR amplification. The EMSA was detected with a Lightshift Chemiluminescent EMSA Kit (Beyotime, 560 GS009, Shanghai, China). The probe and fusion protein were combined in 2× binding buffer for 10 min at room temperature before adding the probe with tag and continuing binding for 25 min at 25 °C. The reaction product was eventually transformed into nylon membranes after 6% PAGE electrophoresis, which was detected by chemiluminescence.

### 4.11. Dual-Luciferase Assays

The coding sequences of ERF3 were inserted into pGreenII 62-SK under the control of the CaMV35S promoter. The promoter of *PeHECT1* was cloned into the pGreenII 0800-LUC reporter vector. The LUC fluorescence signals are detected using in vivo imaging systems after transient transformation of the effector and reporter into tobacco leave for 2 days. The fluorescence emitted from luciferase was observed using a low-light cooled CCD imaging apparatus (Lumazone, PYLoN300B Trenton, America) after a Luciferin (100 μM) spray. The images were then processed and analyzed using ImageJ 1.54k. Additionally, the activities of firefly luciferase (LUC) and renilla luciferase (REN) were measured using the Dual-Luciferase Reporter Assay System (Yeasen, 11406ES60, Shanghai, China).

### 4.12. Quantification and Statistical Analysis

Quantification analyses for all measurements were performed using GraphPad Prism 8. Data are presented as mean ± standard deviation (SD). Statistical significance was assessed using the Student’s *t* test, * *p* < 0.05, ** *p* < 0.01.

## 5. Conclusions

In this study, 16 PeHECT genes were identified in *P. edulis*, categorized into six groups, and mapped to 12 chromosomes. A comprehensive analysis encompassing the evolution, conserved motifs and domains, synteny genome regions, expression patterns, and functions of PeHECT genes was conducted. Notable, *PeHECT1* was found to be responsive to cold and dehydration stress, which is likely attributable to its distinct expression in roots and young shoots. In addition, the ectopic expression of *PeHECT1* in tobacco significantly increased the expression of abiotic stress-related genes. The transcription factor of PeERF3 was directly bound to the promoter of *PeHECT1* and activated the transcription activity. Then, the evolution analysis across various bamboo species indicated that the *PeHECT1* allele in *P. edulis* might represent an ancient variant associated with cold resistance. Overall, these insights contribute to an in-depth understanding of the HECT E3 ubiquitin ligases gene family in *P. edulis* and provide evidence for the potential role of *PeHECT1* in plant stress response.

## Figures and Tables

**Table 1:** Basic characteristic of the PeHECTs.

Gene Name	Gene Locus	CDS (bp)	ORF (aa)	MW (kDa)	pI	Sub-Localization
*PeHECT1*	*PH02Gene13644.t1*	2502	833	93,941.7	8.69	Nucleus.
*PeHECT2*	*PH02Gene17672.t1*	2193	730	83,218.85	6.18	Cell membrane. Chloroplast. Nucleus.
*PeHECT3*	*PH02Gene49527.t1*	11,172	3723	409,025.9	4.9	Nucleus.
*PeHECT4*	*PH02Gene03120.t1*	4260	1419	157,264.32	5.3	Cell membrane. Chloroplast. Nucleus.
*PeHECT5*	*PH02Gene29262.t1*	3093	1030	116,637.61	6.72	Chloroplast. Nucleus.
*PeHECT6*	*PH02Gene39759.t1*	4590	1529	116,726.65	6.49	Cell membrane. Nucleus.
*PeHECT7*	*PH02Gene48467.t1*	10,956	3651	403,204.63	5.03	Nucleus.
*PeHECT8*	*PH02Gene22513.t1*	10,959	3652	403,077.47	5.05	Nucleus.
*PeHECT9*	*PH02Gene15702.t1*	3495	1164	130,482.76	7.47	Nucleus.
*PeHECT10*	*PH02Gene31349.t1*	5385	1794	194,572.66	5.44	Nucleus.
*PeHECT11*	*PH02Gene04390.t1*	3600	1199	134,213.41	5.66	Nucleus.
*PeHECT12*	*PH02Gene44759.t1*	3093	1030	167,934.67	5.17	Nucleus.
*PeHECT13*	*PH02Gene04591.t1*	2577	858	96,687.03	6.88	Cell membrane. Chloroplast. Nucleus.
*PeHECT14*	*PH02Gene25599.t1*	11,160	3719	407,938.91	4.95	Nucleus.
*PeHECT15*	*PH02Gene14896.t2*	5727	1908	206,077.22	5.87	Nucleus.
*PeHECT16*	*PH02Gene19094.t1*	5647	1882	203,207.56	5.94	Nucleus.

**Table 2:** Annotation of co-expression hub genes.

GeneID	Gene Annotation
*PH02Gene00005*	VQ motif
*PH02Gene00040*	Putative uncharacterized protein
*PH02Gene00143*	Rtf2 RING-finger
*PH02Gene00205*	Protein of unknown function
*PH02Gene00288*	Peptidase family C54
*PH02Gene00330*	AMP-binding enzyme C-terminal domain
*PH02Gene00500*	Ion transport protein
*PH02Gene00543*	Lethal giant larvae (Lgl)-like, C-terminal
*PH02Gene00700*	MATH domain
*PH02Gene00792*	HCNGP-like protein
*PH02Gene00831*	UBA/TS-N domain
*PH02Gene00897*	2OG-Fe (II) oxygenase superfamily
*PH02Gene00936*	26S proteasome subunit RPN7
*PH02Gene00944*	RNA recognition motif
*PH02Gene01146*	Phosphomethylpyrimidine kinase
*PH02Gene01178*	G-protein alpha subunit
*PH02Gene01195*	NB-ARC domain
*PH02Gene01229*	Alpha-amylase C-terminal beta-sheet domain

## Data Availability

The original contributions presented in this study are included in the article/Supplementary Materials. Further inquiries can be directed to the corresponding author.

## Supplementary Materials

The supporting information can be downloaded at: https://www.mdpi.com/article/10.3390/ijms252211896/s1.
