# Supplementary material for: Genome-Wide Analysis of HECT E3 Ligases Members in Phyllostachys edulis Provides Insights into the Role of PeHECT1 in Plant Abiotic Stress Response
Source: Int J Mol Sci. 2024 Nov 5;25(22):11896. doi: 10.3390/ijms252211896 (PMC11593785; doi:10.3390/ijms252211896)
Supplement: Supplementary file 1 [file ijms-25-11896-s001.zip › Supplementary table 1.pdf]

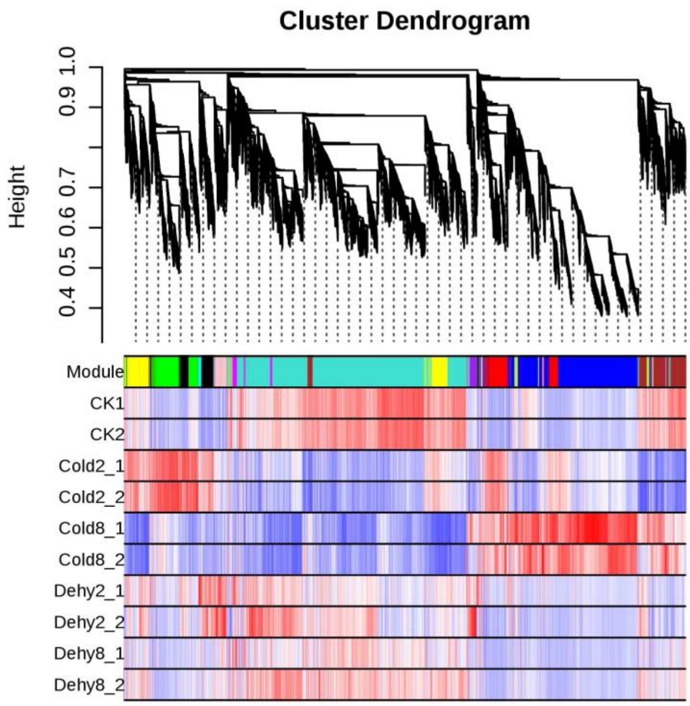

Supplementary Figure S1. Weighted Gene Co-expression Network Analysis (WGCNA) of RNA-seq from *P. edulis* seedlings subjected to cold and dehydration stress. (A) Analysis of module-trait correlations. (B) Analysis of module-sample correlations.

Supplementary Figure S2. Co-expressed network of blue module.

Supplementary Table S1. The primer and probe sequences used in this study.

Primer Name	Primer Sequence F (5′- 3′)	Primer Sequence R (5′- 3′)
PeHECT1-RT	TGCACGAGATGCTCATCGACC	GAAGAACCTTCGTCTGTCGCG
NtSOD-RT	AGCTACATGACGCCATTTCC	CCCTGTAAAGCAGCACCTTC
NtPOD-RT	AAATGGTGGCGCTAGCCGGTG	GCATTGAAGACGTGCCGCTGG
NtCAT-RT	AGGTACCGCTCATTCACACC	AAGCAAGCTTTTGACCCAGA
NtP5CS-RT	GAACGGAGGTTGCTGATGGA	TCCCACTTCGGACTGCTAGA
NtNCED1-RT	AAGAATGGCTCCGCAAGTTA	GCCTAGCAATTCCAGAGTGG
NtLEA5-RT	TTGAATCTGGGGTTTTGGTT	GGAAGCATTGACGAGCTAGG
NtSAMDC-RT	CATTCACATTACCCCGGAAG	AGCAACATCAGCATGCAAAG
NtUBQ-RT	TCCAGGACAAGGAGGGTAT	CATCAACAACAGGCAACCTAG
NtADC1-RT	CTTGCTGATTACCGCAATTTATC	TAGGATCAGCAGCCCCCATAGCC
PeHECT1-GFP	TGTTACTTCTGCAGGAGCTCGTGGCTCAACTCCCTTACACTC	CTCACCATGGATCCGGTACCGAATGCACCAAAGCTGCTGCTCA
PeERF3-AD	gccatggaggccagtgaattcATACGCACACGCCACTTGTAT	atgcccacccgggtggaattcTTACAAGGGACCCCCCCGTCTCT
ProHECT1-AbAi	aaatgatgaattgaaaagcttTCAAATCGACTCAGGTGGTAAA	gtcgacagatccccgggtaccGATACAAGTCAAAGCCCCTCTG

PeERF3-62SK	ccgggctgcaggaattATACGCACA CGCCACTTGTAT	ggtatcgataagctTTACAAGGGACCCCC CCGTCTCT
ProHECT1-0800	GTATCGATAAGCT TCAAATCGA CTCAGGTGGTAAA	TAGAACTAGTGGATC GATACAAGTCAAAGCCCCTCTG
PeERF3-GST	ttccgcgtggatccccggaattcATGGCGCC TAGACCGGCGGAGAAG	ctcgagtcgacccgggaattcCTAGTTCTCCG CCGCCGGCGGCCAG
ProHECT1-probe	TATGGCCGACGTAACGGCCGA CGTCACGGCCGACGAA	TTCGTCGGCCGTGACGTCGGCCGT GATACGTCGGCCATA
